# Practitioner Review: Posttraumatic stress disorder and its treatment in children and adolescents

**DOI:** 10.1111/jcpp.12983

**Published:** 2018-10-23

**Authors:** Patrick Smith, Tim Dalgleish, Richard Meiser‐Stedman

**Affiliations:** ^1^ Department of Psychology Institute of Psychiatry Psychology & Neuroscience King's College London London UK; ^2^ Medical Research Council Cognition and Brain Sciences Unit University of Cambridge Cambridge UK; ^3^ Cambridgeshire and Peterborough NHS Foundation Trust Cambridge UK; ^4^ Department of Clinical Psychology, Norwich Medical School University of East Anglia Norwich UK

**Keywords:** Cognitive therapy, diagnosis, posttraumatic stress disorder, trauma, treatment trials

## Abstract

Important advances in understanding traumatic stress reactions in children and young people have been made in recent years. The aim of this review was to synthesise selected recent research findings, with a focus on their relevance to clinical practice. We therefore address: findings on the epidemiology of trauma exposure and Posttraumatic Stress Disorder (PTSD); recent changes to diagnostic classification; implications for screening and assessment of traumatic stress reactions; and treatment outcome studies including interventions for acute and chronic PTSD, dissemination of effective treatments into community settings, and early interventions. We conclude with recommendations for clinical practice and suggestions for future areas of research.

## Introduction

It has been recognised for nearly 40 years that children and young people may develop Posttraumatic Stress Disorder (PTSD) following exposure to traumatic events (American Psychiatric Association [APA], 1980). Our knowledge and understanding of the epidemiology of trauma exposure and associated mental health problems, of risks for the development and maintenance of PTSD, and of effective treatments for PTSD, have all advanced considerably in recent years. Given the sheer volume of research published since the previous Practitioner Review on PTSD in children and young people (Perrin, Smith, & Yule, 2000), this article does not attempt to provide a comprehensive or systematic review of research findings since then. Rather, we have endeavoured to address selected areas that will be of most relevance to clinical practitioners.

We provide an update on recent findings on rates of trauma exposure and incidence and course of PTSD. The recent re‐formulation to the diagnoses of PTSD in the DSM‐5 (APA, 2013) and ICD‐11 (WHO, 2018) has major implications for all practitioners, and we summarise the differing approaches of the World Health Organization and the APA to diagnosis. Given these changes to diagnostic frameworks, we give an overview of assessment tools and procedures. The second part of this review focuses on recent advances in intervention, asking: Does psychological treatment work and, if so, how? Can treatment be effectively disseminated in front‐line practice? Can early intervention prevent later PTSD? And, do very young children benefit from treatment? We give a summary of psycho‐pharmacotherapy for PTSD, and we critique very recent evidence on the treatment of Complex PTSD. We conclude with a summary and recommendations for practitioners.

## Epidemiology of trauma and PTSD

### Rates of exposure to traumatic events and prevalence of PTSD

An expanding literature speaks to the widespread nature of trauma exposure and PTSD in youth. In their survey of over 6,700 Swiss adolescents, Landolt, Schnyder, Maier, Schoenbucher, and Mohler‐Kuo (2013) found that 56% of 15–16‐year olds had experienced at least one traumatic event that involved ‘actual or threatened death or injury, or a threat to the physical integrity of self or others’ (APA 1980), and that more than a third had experienced multiple traumas. The point prevalence of PTSD was 4.2%, with significantly higher rates in girls. Comparable results were found in the US National Comorbidity Survey for Adolescents (NCS‐A) in which over 6,400 adolescents were asked directly about lifetime exposure to potentially traumatic events that qualified for the DSM‐IV A1 criterion of a traumatic event (McLaughlin et al., 2013). The rate of trauma exposure was just over 60%. Lifetime prevalence of PTSD was 4.7%, with females significantly more likely to meet threshold. Copeland, Keeler, Angold, and Costello (2007) reported that two‐thirds of adolescents in the Great Smoky Mountains Study cohort (*N* = 1420) had been exposed to trauma by the age of 16 years, but the point prevalence estimate for PTSD in this study was <0.5%. These epidemiological studies all show that exposure to trauma is common. Differences in prevalence estimates for PTSD are likely due to differences in assessment methods, sampling methods, and sample sizes; to the lack of developmental sensitivity in diagnostic frameworks; and to the changing DSM criteria for PTSD diagnosis. While prevalence estimates provide broad indicators of morbidity in the community, it is important to note that children with symptoms of PTSD that do not reach threshold for diagnosis may also show significant distress or impairment in functioning (Copeland et al., 2007; Giaconia et al., 1995).

Among trauma‐exposed youth, a wide range of incidence of PTSD has been reported. In their meta‐analysis of 72 cross‐sectional studies from North America, Europe, Australia and Asia, Alisic et al. (2014) found that approximately 16% (95% CI, 11.5–21.5) of trauma‐exposed youth developed PTSD. Females, and youth exposed to interpersonal trauma, were at particular risk. In their meta‐analysis of 27 longitudinal studies, Hiller et al. (2016) estimated PTSD rates of 21% in the acute (1 month) posttrauma phase, spontaneously declining to 15% at 3 months, to 12% at 6 months and to 11% at 1‐year posttrauma. These recent analyses show that a minority of trauma‐exposed young people develop PTSD, and that many recover spontaneously, but spontaneous recovery after 6 months is unlikely.

### Risk and maintaining factors for PTSD

Why do only a minority of trauma‐exposed young people develop persistent PTSD?

Findings from the Great Smoky Mountains Study (Copeland et al., 2007) show that cumulative trauma exposure is relevant. In this longitudinal study of a representative adolescent cohort (*N* = 1420), a lifetime history of multiple trauma exposures strongly predicted higher rates of PTSD symptoms. Trauma type is also relevant. Across multiple studies, higher rates of PTSD are associated with interpersonal or sexual trauma: in Alisic et al.'s (2014) meta‐analysis, exposure to interpersonal trauma was associated with a significantly higher rate of PTSD than exposure to non‐interpersonal trauma (25% and 10% respectively).

Four meta‐analyses have investigated risk factors for persistent PTSD in children and adolescents, each addressing different populations or research questions (Alisic et al., 2014; Cox, Kenardy, & Hendrikz, 2008; Kahana, Feeny, Youngstrom, & Drotar, 2006; Trickey, Siddaway, Meiser‐Stedman, Serpell, & Field, 2012). The largest meta‐analysis to date of risk factors for PTSD in young people (Trickey et al., 2012) includes *N* = 32,238 participants from 64 studies. Despite the broad inclusion criteria and very large sample size, some risk factors were measured in only a handful of studies. This limits the conclusions that can be drawn, but results give a useful overview of the wide‐ranging field. *Negligible or small* increases in risk for persistent PTSD were associated with younger age and minority ethnicity. *Small to medium* effects were observed for female gender, lower IQ, lower socioeconomic status, pre and posttrauma life events, preexisting psychological problems, posttrauma parental mental health, bereavement, and trauma severity. *Large* effects were found for low social support, perceived threat to life, peritraumatic fear, social withdrawal, distraction, thought suppression, presence of other psychopathology, and poor family functioning. Other meta‐analyses have supported an association between parental mental health and child PTSD (Lambert, Holzer, & Hasburn, 2014; Morris, Gabert‐Quillen, & Delahanty, 2012), but there is less evidence of a strong relationship between specific parenting strategies and PTSD (Williamson et al., 2017). As Trickey and colleagues concluded, these studies demonstrate the key role of peri‐traumatic and posttraumatic factors in maintaining persistent PTSD in youth. It is encouraging for practitioners that many of the most significant maintaining factors are potentially modifiable. Cognitive models, summarised below, provide detailed accounts of how these risk and maintaining factors may operate, and how they may be modified in therapy.

### Cognitive models of PTSD

Several cognitive and cognitive‐behavioural models have been proposed for understanding the aetiology and course of PTSD in adults (Brewin, Dalgleish, & Joseph, 1996; Dalgleish, 2004; Ehlers & Clark, 2000; Foa & Kozak, 1986). They describe the role of peri‐traumatic and memory processes, negative appraisals, and maladaptive coping responses in the maintenance of PTSD. For example under Ehlers and Clark's (2000) model, PTSD in adults is characterised by a sense of current threat, which is derived from: (a) individual differences in appraisals of the traumatic event (e.g. ‘nowhere is safe’) and its sequelae (e.g. ‘I am going mad’); and (b) individual differences in the nature of the trauma memory, leading to trauma memories that are more easily triggered by cues in the environment, less accessible to deliberate conscious recall and editing, and more laden with sensory and affective information. The perceived sense of current threat generates a range of maladaptive behavioural and cognitive coping responses usually characterised by avoidance. These coping strategies are maladaptive because they prevent change to negative appraisals and to the nature of the trauma memory; and they can also directly exacerbate PTSD symptoms. Careful consideration of how language, memory, self‐regulation, and social development may influence PTSD in young people is required (Salmon & Bryant, 2002). However, it has been argued that the core elements of cognitive models of PTSD may be applied to youth (Meiser‐Stedman, 2002).

The applicability of cognitive models of PTSD to children and young people has been tested empirically. Several studies demonstrate associations between model‐derived maintaining factors and PTSD symptoms. For example peritraumatic processing style, memory quality, negative appraisals, cognitive avoidance and rumination all show significant associations with PTSD symptoms in cross‐sectional and longitudinal studies in children and adolescents from the age of 8–18 years (Aaron, Zaglul, & Emery, 1999; Duffy et al., 2015; Ehlers, Mayou, & Bryant, 2003; Ellis, Nixon, & Williamson, 2009; McKinnon, Nixon, & Brewer, 2008; Meiser‐Stedman, Dalgleish, Glucksman, Yule, & Smith, 2009; Meiser‐Stedman, Smith, Glucksman, Yule, & Dalgleish, 2007; Meiser‐Stedman et al., 2014; Salmond et al., 2011; Stallard & Smith, 2007). The robust association between negative appraisals and PTSD symptoms has been replicated in a number of studies (Mitchell, Brennan, Curran, Hanna, & Dyer, 2017). Psychological treatments based on cognitive models (see below) explicitly target one or more of these maintaining factors.

## Diagnostic classification

Substantial changes were made to the PTSD diagnosis in the DSM‐5 (APA, 2013). These include: the removal of the disorder from the category of anxiety disorders; changes to the traumatic stressor criteria; the inclusion of an additional symptom cluster to form a four‐factor structure for the diagnosis of PTSD; the use of a unifactorial structure for the related syndrome of Acute Stress Disorder; and the addition of new Preschool and Dissociative subtypes of PTSD. The inclusion of three new PTSD symptoms, and the formation of a new symptom cluster (‘persistent alterations in mood and cognitions’), means that PTSD in the DSM‐5 is more broadly defined than in the DSM‐IV or the ICD‐11. For the first time in the DSM, there is also a new preschool diagnostic subtype of PTSD specifically for children 6 years younger. The rationale for these changes, and the process leading up to them, are described in detail elsewhere (Friedman, 2013).

In contrast to DSM‐5, the new ICD‐11 (WHO, 2018) defines PTSD more narrowly. Symptoms of PTSD are restricted to three core elements (intrusive re‐experiencing, avoidance and arousal), and nonspecific symptoms that are also part of other disorders have been removed. In addition, unlike DSM‐5, ICD‐11 specifies a new diagnosis, Complex PTSD (C‐PTSD), typically arising from multiple, repeated and often interpersonal traumas. C‐PTSD comprises the core elements of PTSD, plus disturbances in three other areas – affect regulation, sense of self and interpersonal relationships. Over the last 3 years, the validity of these major changes to diagnostic criteria, and their implications for clinical practice with children and young people, have been investigated.

Because the ICD and DSM frameworks differ substantially, it is possible that rates of PTSD among trauma exposed young people will differ according to which diagnostic framework is used. Danzi and La Greca (2016) examined this issue directly in hurricane‐exposed children. Overall rates of PTSD were similar for both diagnostic frameworks. However, there was low agreement across the frameworks, with only 38% of children identified by both. ICD‐11 identified children with more severe core PTSD symptoms, whereas DSM‐5 identified children with many noncore symptoms of PTSD. This divergence in identified cases is not unique to children (e.g. see O'Donnell et al., 2014; Stein et al., 2014 for adults), but it clearly has potential to cause confusion in both clinical practice and research. It is recommended that future research uses measures which assess symptoms from both diagnostic frameworks.

Is the underlying structure of PTSD symptoms best accounted for by the DSM‐5 four‐factor model, or by the simpler ICD‐11 three‐factor proposal? Direct comparisons of the factor structures proposed in the DSM‐5 and ICD 11 have not yet been reported for young people. A variety of latent factor structures have received empirical support, including 4‐, 5‐ and 7‐factor models (e.g. Elhai et al., 2013; Liu, Wang, Cao, Qing, & Armour, 2016; Yang et al., 2017), none of which map directly onto the DSM or the ICD frameworks. Model‐fitting using confirmatory factor analysis and related approaches such as latent class analysis can provide important information about the validity of these new diagnostic frameworks, and further work is needed to bring clarity to the area.

The implications for young people of the major overhaul in the DSM‐5 to the Acute Stress Disorder (ASD) criteria have been examined in two recent studies. McKinnon, Meiser‐Stedman et al. (2016) found that DSM‐5′s unidimensional structure to ASD provided a poor fit for early symptoms of trauma exposed children and young people. Symptom clustering was clearly present in the first month after trauma exposure. Kassam‐Adams et al. (2012) showed that the 8‐symptom requirement for DSM‐5 ASD resulted in most children who showed impairment in the first month posttrauma missing out on a diagnosis of ASD. These authors suggest that to maximise sensitivity, a lower symptom threshold of three or four symptoms would be more appropriate for children and young people. The implication for practitioners is that when a child or adolescent presents with three or four ASD symptoms in the acute post trauma period, follow‐up and possible intervention should be considered, regardless of whether formal diagnostic criteria for ASD are met.

The new DSM‐5 diagnosis for children of 6 years and younger is welcomed. It is based on several years of work which showed that very young trauma‐exposed children who presented with significant symptoms, distress and impairment were missing out on DSM‐IV diagnoses (see Meiser‐Stedman, Smith, Glucksman, Yule, & Dalgleish, 2008; Scheeringa, 2011; Scheeringa, Zeanah, & Cohen, 2011). DSM‐5 provides developmentally appropriate criteria that will help practitioners to identify children in need of intervention, and should enhance research progress for this population (de Young, Kenardy, Cobham, & Kimble, 2011).

Constructs approximating Complex PTSD (C‐PTSD) have a long history (see Terr, 1991), but only two studies to date have formally investigated the ICD‐11 C‐PTSD construct in children and adolescents. In a clinically referred sample, Sachser, Keller, and Goldbeck (2017) found that PTSD and C‐PTSD were empirically distinguishable: latent class analysis revealed just two distinct classes, representing the two disorders. In a community sample of trauma‐exposed adolescents and young adults, Perkonigg et al. (2016) also found evidence for latent classes that reflected the distinct symptom profiles of PTSD and C‐PTSD. Young people who have been exposed to trauma can certainly present with disturbances in affect regulation, sense of self and interpersonal relationships. Future research will help to clarify whether these additional symptoms imply a qualitatively distinct disorder, or are an indication of a particularly severe form of PTSD (Bryant, 2012; Resick et al., 2012) or indeed whether the very question of PTSD as a diagnosis should be revisited in the light of interesting developments in the dimensional classification of youth and adult mental health (see Kotov et al., 2017; and Cuthbert & Insel, 2013, for overviews).

## Assessment

It is important to conduct a thorough and structured assessment of trauma exposure and PTSD symptoms. Not completing such assessments for fear of creating distress may represent a gross disservice to child and adolescent clients. A thorough assessment may lead to an offer of treatment and, if handled sensitively, can be therapeutically beneficial through the appropriate recognition and normalisation of the client's experience. Assessing posttraumatic stress need not be a source of significant distress; indeed, young people may be more disturbed if their disclosure of trauma exposure is not followed up by practitioners. It is common for children to try to hide their distress from their parents, for fear of upsetting them, and so interviewing children and adolescents separately from their parents or caregivers (if the child allows such separation) is advisable. Parents will provide valuable insight into how children are coping, but parental report of symptoms should be treated with some caution: parents may under‐report symptoms if children have successfully hidden them, or may over‐report symptoms in their child if they are symptomatic themselves (e.g. Meiser‐Stedman et al., 2007; Stover, Hahn, Berkowitz, & Im, 2010).

There is a variety of freely available structured interviews and questionnaires for the assessment of posttraumatic stress and PTSD in youth. Several assessment tools are available for assessing according to the DSM‐5, but no ICD‐11‐oriented tools exist yet.

### Interview schedules

The Clinician Administered PTSD Scale for Children and Adolescents (CAPS‐CA) is a structured interview for diagnostic assessment of PTSD in 8–15 year olds. It is one of the most frequently used interviews in published trials (see below). It has been adapted from its DSM‐IV format (Nader et al., 1996) to accommodate the DSM‐5 diagnoses, and has been simplified so that it only involves symptom frequency ratings (Pynoos et al., 2015). In dropping the intensity ratings, the authors have produced a more child‐friendly and easily administered instrument that will be useful for practitioners. The CAPS‐CA has shown good internal consistency (Cronbach's alpha .75–.82), excellent interrater reliability (ICC = 0.97), and adequate convergent validity (*r* = .51 against the CPTSD‐RI; see review by Leigh, Yule, & Smith, 2016). The UCLA PTSD Reaction Index (UCLA PTSD‐RI; Steinberg, Brymer, Decker, & Pynoos, 2004) is an interview schedule intended for 6–18 year olds. It has shown excellent internal consistency (Cronbach's alpha = .90) and test–retest reliability (*r* = .84), good convergent validity (*r* = .75), and is relatively brief to administer (Steinberg et al., 2013). A DSM‐5 version is undergoing testing. The Children's PTSD Inventory (CPTSDI; Saigh et al., 2000), demonstrates excellent internal consistency (Cronbach's alpha = .95), interrater reliability (ICC = .98) and test–retest reliability (kappa = .91; Saigh et al., 2000); and good convergent validity (*r* = .70; Yasik et al., 2001), but a DSM‐5 version has not yet been produced. The widely used Child PTSD Symptom Scale (CPSS; Foa, Johnson, Feeny, & Treadwell, 2001; see below) has been adapted to an interview‐version (Foa, McLean, Capaldi, & Rosenfield, 2013), with a DSM‐5 variant now available (CPSS‐5‐I; Foa, Asnaani, Zang, Capaldi, & Yeh, 2018). The CPSS‐5‐I shows excellent internal consistency (Cronbach's alpha .92), test–retest reliability (*r* = .93) and interrater reliability (kappa = 1; Foa et al., 2018). At the time of writing it is the only interview measure for children and young people based on the DSM‐5 that has published psychometric properties (Box 1).

Box 1 **Assessment measures**

Assessment of trauma‐exposed young people should routinely employ valid and reliable interview and questionnaire measures of PTSD and other common problems.

**Interviews**

Clinician Administered PTSD Scale for Children and Adolescents (CAPS‐CA) (Pynoos et al., 2015)
UCLA PTSD Reaction Index (UCLA PTSD‐RI) (Steinberg et al., 2004)
Children's PTSD Inventory (CPTSD‐I) (Saigh et al., 2000)
Child PTSD Symptom Scale – Interview (CPSS‐5‐I) (Foa et al., 2018)

**Self‐report questionnaires**

Children's Revised Impact of Event Scale (CRIES‐8) Perrin et al., 2005)
Child Trauma Screening Questionnaire (CTSQ) (Kenardy et al., 2006)
Child PTSD Symptom Scale (CPSS) (Foa et al., 2018)
Child and Adolescent Trauma Screen (CATS) (Sachser, Berliner et al., 2017)

### Self‐report questionnaires

Self‐report questionnaires can serve a number of purposes. When screening a large number of children in a short space of time to identify those with probable PTSD (e.g. after a natural disaster), measures that are sensitive and specific while being quick to administer and score are required. The CRIES‐8 is a brief, eight‐item measure of intrusion and avoidance symptoms (Perrin, Meiser‐Stedman, & Smith, 2005), with robust psychometric properties and well‐established cut‐points. It has been translated into several languages (see Children and War Foundation, www.childrenandwar.org), is very quick to administer, and is likely impervious to further refinement of the different diagnostic systems. Its main limitation is that it does not address some core re‐experiencing symptoms, but the extended 13‐item version does address hyperarousal symptoms. Alternatively, the 10‐item Child Trauma Screening Questionnaire (CTSQ; Kenardy, Spence, & Macleod, 2006) is also very quick to administer and is efficient in identifying PTSD cases.

Questionnaires that are used as part of a detailed clinical assessment should be reliable, valid, and sensitive to clinical change. When used appropriately, these questionnaires can help clinicians to normalise symptoms, and can provide a useful starting point for discussions with avoidant children. The Child PTSD Symptom Scale (CPSS; Foa et al., 2001, 2018) is widely used with young people aged 8–15 years old. The measure demonstrates excellent internal reliability and test‐re‐test reliability, is sensitive to change during and after treatment, and has been updated to address all DSM‐5 PTSD symptoms. Good convergent validity has been found in a number of studies (Nixon et al., 2013). The newly developed Child and Adolescent Trauma Screen (Sachser, Berliner et al., 2017) shows considerable promise as a measure of DSM‐5 PTSD symptoms and includes a trauma exposure screen.

The Child Post‐Traumatic Cognitions Inventory is a useful index of negative trauma‐related beliefs among 7–17 year olds. In addition to its standard 25‐item version (Meiser‐Stedman, Smith et al., 2009), a 10‐item version for easier use in the clinic has been developed (McKinnon, Smith et al., 2016), and numerous translations of the measure are available (de Haan, Petermann, Meiser‐Stedman, & Goldbeck, 2016; Diehle, de Roos, Meiser‐Stedman, Boer, & Lindauer, 2015).

For the assessment of the new DSM‐5 PTSD diagnosis for children 6 years old and younger, Scheeringa & Zeanah (2005) have developed a structured interview and a questionnaire measure based on parent or caregiver report. The Pediatric Emotional Distress Scale (Saylor, Swenson, Reynolds, & Taylor, 1999) also uses caregiver report, and has been shown to have useful properties as a screening tool for young children.

Validated tools for assessing the Complex PTSD symptoms in ICD‐11 have yet to be established. The development of such a tool for children and young people should be a priority for research in this area. To date, researchers have addressed this difficulty using multiple measures.

In summary, we recommend a broad‐based (including symptoms of anxiety, depression and other problems), multi‐modal (interview and questionnaire), multi‐informant (child and carer) approach to assessment. The interview schedules mentioned above are all suitable for clinical practice. The CRIES‐8 (Perrin et al., 2005) has many benefits as a screening tool; the CPSS questionnaire (Foa et al., 2001, 2018) is recommended for detailed clinical assessment, and as an outcome tool.

## Interventions

### Psychological interventions

In this selective review, we have chosen to focus on trauma‐focused interventions because they are the most thoroughly investigated of all psychological treatments. These include Trauma‐Focused CBT (TF‐CBT, e.g. Cohen, Deblinger, Mannarino, & Steer, 2004), Prolonged Exposure (PE, e.g. Foa et al., 2013), and Cognitive Therapy for PTSD (CT for PTSD, e.g. Smith et al., 2007; Dalgleish et al., 2015; Meiser‐Stedman, Smith, McKinnon et al., 2017). These interventions are based on empirically supported theories of persistent PTSD, and aim to reverse one or more of the maintaining factors referred to above. There is considerable overlap in treatment components of these various forms of trauma‐focused intervention. Typical treatment components include: psychoeducation about PTSD; development of a shared treatment rationale; behavioural activation; relaxation training; imaginal exposure to the trauma memory (imaginal reliving); cognitive restructuring and memory updating and planned exposure to trauma triggers and reminders. Practically, differences between these CBT treatment approaches lie in the relative weight given to particular aspects of intervention. For example PE places relatively more emphasis on relaxation training and imaginal exposure, whereas CT for PTSD places relatively more emphasis on cognitive restructuring and memory updating. A course of trauma‐focused treatment lasts from 10 to 20 weekly sessions, is usually delivered individually to the young person, and will almost always involve parents or carers.

It is now‐well established that trauma‐focused interventions for PTSD in children and young people are highly efficacious. A number of recent reviews and meta‐analyses summarise the available evidence. Morina, Koerssen, and Pollet's (2016) meta‐analysis included 39 RCTs of psychological interventions for paediatric PTSD. Trauma‐Focused CBT was the most researched form of intervention, and produced the largest effect sizes (Hedge's *g *=* *1.44 when compared to wait list; *g *=* *0.66 when compared with an active control condition). Gutermann et al's. (2016) larger meta‐analysis included 135 studies of psychological interventions for symptoms of paediatric PTSD, and also found that trauma‐focused CBT interventions (broadly defined as TF‐CBT, PE, and CT for PTSD) showed the largest effect sizes (Hedge's *g *=* *1.39). These recent meta‐analyses are consistent with Gillies, Taylor, Gray, O'Brien, and D'Abrew's (2012) Cochrane review, which reported that CBT showed the best evidence of effectiveness for up to a year following treatment, compared to other interventions such as psychodynamic psychotherapy and supportive counselling. Previous reviews (e.g. Silverman et al., 2008; Dalgleish, Meiser‐Stedman, & Smith, 2005) came to similar conclusions.

Methodological limitations to some of the RCTs included in the above meta‐analyses should be noted. Morina et al.'s (2016) formal assessment indicated a satisfactory level of quality for most of the constituent trials. However, only a minority of trials used a reliable semi‐structured interview to assess PTSD, or employed blind assessors for postintervention assessments. Most studies did not report follow‐up outcomes; and of those that did, follow‐up beyond 6 months was rare. Less than half of included trials reported an ‘Intent to Treat’ (ITT) analysis. Sample size is also a concern in some RCTs. Overall, *N* = 4184 participants across 39 RCTs were included in Morina et al.'s (2016) meta‐analysis. However, the size of individual studies varied greatly from *N* = 24 in two small pilot studies, to *N* > 300 in five studies of classroom‐based interventions. In the 22 studies that evaluated TF‐CBT, sample sizes ranged from *N* = 24 to *N* = 180. Despite small sample sizes in some initial trials, it is encouraging for the field that large effect sizes were also detected in much larger dissemination effectiveness trials.

In summary, in a number of RCTs, trauma‐focused interventions show medium to large effects on reducing PTSD symptoms and comorbid conditions. These effects appear durable, and relapse is rare. These findings apply to children and young people across the age range from 8 to 18 years old who have been exposed to a range of traumatic events including sexual abuse, physical abuse, and single event traumas such as assaults and accidents.

The weight of trial evidence is clearly in favour of trauma‐focused CBT interventions, but there is growing evidence that Eye Movement Desensitisation and Reprocessing (EMDR) is promising for children and young people with PTSD. In this short‐term treatment, clients recall a traumatic event while engaging in saccadic eye movements or other forms of dual attention such as tapping (Shapiro, 2001). To date, seven RCTs have investigated EMDR for young people with PTSD (Ahmad, Larsson, & Sundelin‐Wahlsten, 2007; Chemtob, Nakashima, & Carlson, 2002; de Roos et al., 2011, 2017; Diehle, de Roos et al., 2015; Diehle, Opmeer, Boer, Mannarino, & Lindauer, 2015; Jaberghaderi, Greenwald, Rubin, Zand, & Dolatabadi, 2004; Kemp, Drummond, & McDermott, 2010). Sample sizes tend to be small (Ns < 53 in 6 out 7 studies), and only two studies used diagnostic interviews to assess PTSD. Results from the four trials which compared EMDR to trauma‐focused CBT suggest that the interventions are equally effective. However, inspection of the pre‐post effect sizes suggests that TF‐CBT in these trials was not as efficacious as in other trials of TF‐CBT. Given the relative scarcity of rigorous evaluations of EMDR, further work is required. The National Institute for Health and Care Excellence (NICE, 2005, 2018) and the WHO (2013) recommend EMDR as an option for use with adults. The current NICE guideline (NICE, 2018), out for consultation at the time of writing, states that EMDR should be considered for children and young people with persistent PTSD if they do not engage with or respond to TF‐CBT.

In the context of these positive findings on efficacy, we address further questions about treatment below (Box 2).

Box 2 **Interventions**

*Trauma‐Focused CBT interventions* are recommended by National Institute for Health and Care Excellence (2018) as the first‐line treatment for persistent PTSD in children and young people. These interventions include: Trauma‐Focused CBT (TF‐CBT), Prolonged Exposure for PTSD (PE), and Cognitive Therapy for PTSD (CT‐PTSD).

*Eye Movement Desensitization and Reprocessing* (EMDR) is recommended by National Institute for Health and Care Excellence (2018) as a treatment option if young people to do not engage in, or do not respond to, Trauma‐Focused CBT.

*Medication* is not recommended for the prevention or treatment of PTSD in children and young people.

### How does psychological treatment work?

A clear implication of Ehlers and Clark's (2000) model (above) is that modifying negative misappraisals during trauma‐focused therapy will lead to reductions in PTSD symptoms. This has been tested directly in five recent RCTs, using mediation analysis. Changes in dysfunctional appraisals were shown to partially mediate the effect of CT for PTSD in the treatment of early (Meiser‐Stedman, Smith, McKinnon et al., 2017) and persistent PTSD (Jensen, Holt, Mørup Ormhaug, Fjermestad, & Wentzel‐Larsen, 2018; Pfeiffer, Sachser, de Haan, Tutus, & Goldbeck, 2017; Smith et al., 2007). It is also interesting to note that in Prolonged Exposure, changes in negative trauma‐related cognitions were found to mediate the effect of treatment on PTSD symptoms (McLean, Yeh, Rosenfield, & Foa, 2015). These findings are in line with adult studies which have shown changes in dysfunctional appraisals to be a core treatment mechanism of trauma‐focused therapy (Kleim et al., 2012). The finding that changes to misappraisals do not fully mediate the effect of treatment suggests that other mechanisms are also operating. Meiser‐Stedman, Smith, McKinnon et al. (2017) found that, as predicted by theories of PTSD maintenance, reductions in safety‐seeking behaviour (a form of avoidance) also partially mediated the effect of CT for PTSD. Further research on treatment mechanisms has potential to identify which processes are important during therapy. This in turn can inform the refinement of therapies so that they are efficient, as well as efficacious. From an individual case point of view, these findings imply that patients will achieve maximum benefit from therapy when practitioners focus on reversing these theoretically derived, empirically supported, maintaining factors.

### Can treatment be disseminated without loss of effectiveness?

Many of the RCTs included in the meta‐analyses above were modest in size, and were carried out in specialist university‐based research clinics. A key question for practitioners is whether the positive findings from these RCTs can be translated into regular, busy, front‐line clinical practice. Two large effectiveness studies have been carried out recently.

Jensen et al. (2014) implemented a randomised effectiveness study in community clinics in Norway. TF‐CBT was delivered by community clinicians with a range of professional backgrounds (psychologists, psychiatrists, educational therapists and social workers), and compared to an active comparison condition, Treatment as Usual (TAU). *N* = 156 young people (10–18 years old) took part. After TF‐CBT, 79% were free of PTSD diagnosis, compared to 55% who received TAU. TF‐CBT was also associated with reduction in depression and improvements in general mental health. Treatment gains were maintained at follow‐up 18 months later (Jensen, Holt, & Ormhaug, 2017). Goldbeck, Muche, Sachser, Tutus, and Rosner (2016) carried out a randomised effectiveness study in community mental health clinics in Germany. Treatment was delivered by psychologists, psychiatrists and social workers. Young people (7–17 years old) were randomised to receive TF‐CBT (*N* = 76), or to be on a Waiting List (*N* = 83). TF‐CBT was found to be significantly superior to WL in the treatment of PTSD, and also in improvements to depression and anxiety. In both of these effectiveness studies, the inclusion criteria were broad, and study therapists had heterogeneous professional backgrounds and levels of training. The dropout rates for TF‐CBT were similar in each study, at around 15%. Both studies showed large within‐group effect sizes on PTSD symptoms (*d* = 1.49 and *d* = 1.51) that were similar to the effect sizes reported in prior efficacy studies.

### New approaches to treatment delivery

The work summarised above shows that when delivered face‐to‐face in specialist research clinics, or in routine community clinic settings, trauma‐focused cognitive behavioural interventions are effective. However, there may be barriers to accessing these effective treatments. In some health‐care settings, there are simply too few specially trained practitioners to deliver trauma‐focused interventions (Royal College of Psychiatrists, 2013). When specialist services are available, perceived stigma may deter some families from accessing them. There may be practical barriers such as time commitment or cost. New models of service delivery may therefore be needed. Two new approaches have been investigated recently.

First, a stepped care approach has been developed and trialled by Salloum et al. (2016). Under this model, Step 1 is a parent‐led treatment. For the first 6 weeks, parents provide most of the treatment for their children at home, supported by weekly phone calls, web‐based psychoeducation, and three clinic‐based sessions. At the end of Step 1, children who require further treatment are offered nine conventional clinic‐based TF‐CBT sessions. Salloum et al.'s (2016) RCT compared stepped‐care TF‐CBT to standard clinic‐based TF‐CBT, for 3–8 year olds with symptoms of PTSD. The stepped care approach was acceptable to parents. There were no significant differences in treatment response between the two conditions. Of children allocated to stepped‐care, 22/32 (69%) responded to Step 1, and did not require further face‐to‐face TF CBT. The stepped care approach was significantly less costly than the standard approach. To date, this parent‐led approach has been evaluated with younger children only, and it is an open question whether it would work for adolescents.

Second, a number of research groups have investigated online delivery of trauma‐focused interventions. This follows promising work with adults, which has shown that online TF‐CBT is acceptable to patients, and is associated with large pre‐post treatment effect sizes on symptoms of PTSD, depression and anxiety (Knaevelsrud & Maercker, 2007, 2010; Lewis et al., 2017; Litz, Engel, Bryant, & Papa, 2007; Spence et al., 2011). Importantly, in these studies, therapist time was less than a quarter of that needed in standard face‐to‐face therapy. Ruggiero et al. (2015) developed a web‐based intervention, for disaster affected adolescents and their parents. A modular approach was used to provide psychoeducation about PTSD symptoms, depression and substance misuse using appealing graphics, animations and videos. In a large RCT including *N* = 2,000 adolescents who had been exposed to a tornado, the intervention was associated with improvements in adolescents’ PTSD and depressive symptoms at 12‐month follow‐up. Marsac, Donlon, Winston, and Kassam‐Adams (2013), and Marsac et al. (2015) developed an online intervention, *Coping Coach,* to prevent PTSD among young children exposed to acute medical events. The intervention aims to promote adaptive appraisals, decrease avoidance and promote social support. Children (8–12 years old) progress through an interactive game in which they earn points. In a pilot RCT, Kassam‐Adams et al. (2016), showed that most children who were offered the intervention used it, and that half of them completed it. However, children who were offered *Coping Coach* reported similar levels of PTSD symptoms at 6‐ and 12‐week follow‐up, as children who were randomly allocated to a waiting list. Outcomes in this small pilot trial are difficult to interpret because of baseline differences between groups. Digital health interventions have enormous potential for wide dissemination of effective treatment for PTSD in children and adolescents. Interventions appear to be acceptable to young people and families, and feasible to deliver, but currently clinical outcomes are mixed.

### Can early intervention prevent PTSD?

Given that PTSD is one of the few psychiatric diagnoses to specify an external aetiological agent, it should be possible to develop early interventions to prevent persistent PTSD among trauma‐exposed children and young people. Several trials have tested this directly.

Stallard et al.'s (2006) RCT compared a single‐session of debriefing to a control condition (talking about a neutral topic), both delivered within a month of exposure to a road traffic accident, to 158 children and adolescents (7–18 years old). At 8‐month follow‐up, all children improved, but there was no significant difference between the groups on PTSD symptoms. Kenardy, Thompson, Le Broque, and Olsson's (2008) RCT compared provision of psychoeducation in a booklet, to a no‐intervention control. The psychoeducation booklet was delivered to children within 3 days of hospital admission following accidental injury. Children were followed up at 1 and 6 months. There were no significant differences on child PTSD symptoms at either time point. Zehnder, Meuli, and Landolt's (2010) RCT compared a single‐session early psychological intervention to standard care for 99 children (7–16 years old) who had been involved in a road traffic accident 10 days previously. Children were followed up at 2 and 6 months. There were no significant differences on child PTSD symptoms at either time point.

Negative findings for universally delivered single‐session interventions have stimulated the development of other approaches. Berkowitz, Stover, and Marans (2011) employed a screen‐and‐intervene approach, using the 4‐session Child and Family Traumatic Stress Intervention (CFTSI). The emphasis in CFTSI is on psycho‐education, improving family communication, and teaching CBT skills relevant to the child's individual needs. In an RCT including 112 children (7–17 years old) who reported at least one new PTSD symptom after trauma exposure, children who received the CFTSI intervention within a month of trauma exposure reported significantly fewer symptoms of PTSD and anxiety, and were significantly less likely have a PTSD diagnosis, when followed up at 3 months (Berkowitz et al., 2011). These results are encouraging, especially considering the comparison group involved some highly credible therapy components (i.e. psychoeducation, relaxation techniques). However, it is important to note that the outcome assessments were undertaken by nonblind interviewers. Kramer and Landolt (2014) also employed a screen‐and‐intervene approach, using a 2‐session CBT intervention, delivered at home 10–16 days after trauma exposure. This intervention was compared to treatment‐as‐usual for 108 children (2–16 years old) who had screened positive for significant symptoms of PTSD following a road traffic accident or suffering a burn injury. Children were followed up at 3 and 6 months. Among preschool children, there was no effect on any outcome. Among school‐age children, those in the early intervention group showed significantly fewer anxiety problems at 3 months, but not at 6 months. There was a nonsignificant trend for reduction in PTSD intrusion symptoms at 3 months among the school age children, but no effect on diagnosis of PTSD.

Overall, findings on preventive interventions are mixed. Among trauma‐exposed adults, there is evidence that brief, universally delivered early interventions can slow down natural recovery in individuals who are highly symptomatic at baseline. In a well‐known RCT (Mayou, Ehlers, & Hobbs, 2000) that compared a single session of debriefing to no‐intervention for adult survivors of traffic accidents, this negative effect was evident at 4‐month posttrauma, and persisted 3 years later. Such findings led to a clear recommendation in a Cochrane review (Rose, Bisson, Churchill, & Wessely, 2002) that early intervention should not be routinely delivered to all trauma‐exposed adults. However, among young people, there is no evidence that early intervention is harmful in this way. Equally, there is no evidence that a single‐session intervention, delivered universally to all trauma exposed children within the first few days after trauma, is effective. However, multisession early interventions, targeted at children who report significant PTSD symptoms, show clear promise.

### Do treatments work for very young children?

Participants in most of the RCTs cited in the reviews and meta‐analyses referred to above were of school age, and usually 8 years and older. Given the persistence of PTSD for several years if left untreated in very young children (younger than 8 years old) (Meiser‐Stedman, Smith, Yule, Glucksman, & Dalgleish, 2017), there is a need to develop age‐appropriate interventions for this age group, and to evaluate them rigorously. Important adaptations to standard trauma‐focused CBT protocols are needed when treating PTSD in young children. These may include: greater involvement of carers throughout including joint carer‐child sessions; behaviour management training for carers; skills teaching in arousal reduction for children; and use of developmentally engaging methods to construct trauma narratives (see Scheeringa, 2016). A number of promising and informative case examples have been published (Goodall et al., 2017; Scheeringa et al., 2007). To date, there has been one pilot RCT evaluating developmentally adapted TF‐CBT in younger children with PTSD symptoms (Scheeringa, Weems, Cohen, Amaya‐Jackson, & Guthrie, 2011). Compared to wait list, TF‐CBT for 64 children (3–6 years old) demonstrated a large effect size, with treatment gains being maintained at 6 month follow‐up. These findings are encouraging, but given the limited outcome research with younger children to date, further work is clearly needed (Dalgleish et al., 2015).

### Does Complex PTSD in young people require a new treatment approach?

With the recent publication of ICD‐11, issues around the treatment of Complex PTSD in children and young people are likely to take centre stage. As discussed above, Complex PTSD is a variant of PTSD. The diagnosis may be most applicable to individuals who have been exposed to multiple repeated, interpersonal traumas, although it can be made following exposure to a single event trauma. The construct includes core symptoms of PTSD, plus one symptom from each of three additional domains, namely: (a) affective dysregulation; (b) negative self‐concept, and (c) relational difficulties (Maercker et al., 2013; WHO, 2018).

The key question about treatment is whether these additional symptom domains require the addition of new treatment components. Specifically, do individuals who have difficulties with affect dysregulation require intervention to improve emotional regulation, prior to engaging in trauma‐focused work?

The Expert Consensus Treatment Guidelines for Complex PTSD in Adults (Cloitre et al., 2012), issued by ISTSS Complex Trauma Task Force, recommend such a phased approach to treating CPTSD in adults. Phase 1 is intended to improve emotional stabilisation by reducing self‐regulation problems and by improving emotional social and psychological competences. Phase 2 is trauma focused (in effect, trauma memory focused), as in standard trauma‐focused interventions. Phase 3 is an integration phase, intended to consolidate treatment gains. An example of this phased approach is given by Cloitre et al. (2010). These authors describe an initial preparatory phase of *skills training in affect and interpersonal regulation* (STAIR), followed by a second phase of exposure‐based treatment. In an RCT including *N* = 104 women with PTSD related to child abuse, this phased skills‐to‐exposure treatment outperformed treatments which omitted either the skills‐based phase, or the exposure‐based phase.

However, the need for an initial stabilisation phase prior to memory‐focused treatment has been questioned. de Jongh et al. (2016) provide a detailed review and critique of PTSD treatment outcome studies in adults. They note that no studies have yet directly compared standard trauma‐focused treatments to phased oriented treatments for PTSD. They suggest that affect dysregulation may be a symptom of PTSD that improves after trauma‐focused treatment, rather than needing direct symptomatic intervention in itself. On this view, a phased‐based approach may run the risk that patients are delayed in receiving effective conventional treatment.

We have referred here to adult work because few child or adolescent studies have directly addressed this issue. However, recent secondary analysis (Sachser, Keller et al., 2017) of previous trial data (Goldbeck et al., 2016) suggested that children who met criteria for C‐PTSD (defined using latent class analysis) responded as well to TF‐CBT as did children with PTSD. The form of TF‐CBT used in that trial was initially developed by Cohen et al. (2004) to treat symptoms of posttraumatic stress and other trauma‐related difficulties among young people who had experienced sexual abuse. Multiple, repeated, interpersonal trauma such as childhood sexual abuse is implicated in the development of what is now understood as C‐PTSD. Cohen's treatment includes affect regulation skills teaching for young people in addition to memory‐focused work, and has consistently demonstrated powerful effects in treating PTSD and broader difficulties (e.g. Cohen et al., 2004; Cohen & Mannarino, 2017). Young asylum seekers and refugees will often have experienced or witnessed repeated interpersonal traumas. It has long been recommended that this population is offered a flexible, phased‐based approach which incorporates skills teaching and memory‐focused work (e.g. Ehntholt & Yule, 2006). Given the scarcity of studies to date, further work is needed to evaluate which treatment components are necessary for the effective and efficient treatment of C‐PTSD in young people.

## Pharmacotherapy for PTSD in children and young people

Few randomised controlled trials of medication for paediatric PTSD have been carried out. Cohen, Mannarino, Perel, and Staron (2007) compared TF‐CBT plus sertraline (an SSRI) to TF‐CBT plus placebo for the treatment of PTSD in *N* = 24 young people (10–17 years old) who had developed PTSD following sexual abuse. After treatment, there wa s no significant difference in PTSD symptom severity between the two groups. Robb, Cueva, Sporn, Yang, and Vanderburg (2010) compared sertraline alone to placebo alone for the treatment of PTSD in *N* = 131 young people with PTSD symptoms. There was no significant difference between the two groups on PTSD symptoms after treatment. Robert et al.'s (2008) RCT trialled fluoxetine (an SSRI) against imipramine (a TCA) and placebo in the treatment of acute stress disorder. There was no significant difference between the three groups on symptoms of ASD after a short 1‐week treatment period. Findings from open‐label uncontrolled case series and single case reports have reported some beneficial effects on PTSD symptoms in children after use of antipsychotics (risperidone, quetiapine), mood stabilisers (carbamazepine) and anti‐adrenergic agents (guanfacine, clonidine, propranolol). Given the design limitations and generally small sample sizes, these findings must be treated with caution. Finally, Scheeringa and Weems (2014) report on the use of D‐cycloserine to enhance exposure therapy in the treatment of children with PTSD. Fifty‐seven young people (7–18 years old) with PTSD were allocated to TF‐CBT with D‐cycloserine, or to TF‐CBT with a placebo. There were no significant differences between the groups on PTSD symptoms at the end of treatment, nor on rate of improvement during treatment.

Practice guidelines from the UK (NICE, 2005, 2018), the US (AACAP, 2010), and the International Society for Traumatic Stress Studies (Foa, Keane, Friedman, & Cohen, 2010) all recommend trauma‐focused talking therapies as the first‐line treatment for PTSD in young people. In the UK, NICE (2018) states, ‘do not offer drug treatments for the prevention or treatment of PTSD in children and young people aged under 18 years’. In the United States, the American Academy of Child and Adolescent Psychiatry recommends that, ‘SSRIs can be considered for the treatment of children and adolescents with PTSD’. The cautious approach in these professional guideline recommendations reflects the very small evidence base, and the lack of strong evidence for medications in the few trials that have been conducted.

## Summary and recommendations

While trauma exposure is common, persistent PTSD occurs in a minority of exposed young people, many of whom will recover without any professional help. Cognitive models help us to understand why some children develop persistent PTSD. Key changes have been made recently to the diagnosis of PTSD for young people, and the development and testing of assessment instruments is catching up with these changes: practitioners will benefit from using broad‐based, multiinformant, developmentally appropriate tools that are practical to use and acceptable to children and families. Theory‐based, trauma‐focused psychological therapies are efficacious for treating persistent PTSD, and recent work has shown how these treatments can be used by practitioners in community clinics without loss of effectiveness. Despite this, health‐care providers often do not have capacity to offer these effective treatments at the level required. Few trials of face to face therapy for preschool children with PTSD have been completed, but developmentally adapted trauma‐focused interventions are likely to be helpful. Progress towards developing early interventions to prevent persistent PTSD among trauma‐exposed children and young people has been slow. There is a lack of compelling evidence for pharmacotherapy for PTSD in children and young people.

### Recommendations

Although trauma‐focused interventions are efficacious, most young people with PTSD are not offered an evidence‐based intervention. There is a need for strategies which enable evidence‐based treatments to be made much more widely available for young people. One strategy is to offer specialist training and supervision to practitioners so that PTSD is better detected, and evidence‐based face‐to‐face therapy is routinely offered. A complementary strategy is to develop stepped care approaches, or e‐health approaches, as described above. Such strategies should be carefully evaluated using an implementation science framework. Large‐scale effectiveness studies present opportunities for examining combined mechanisms of treatment effect (‘how do treatments work?’), and moderators of treatment effect (‘for whom do treatments work best?’). Evidence on mediators and moderators can help to refine treatment components, and can inform the way that systems are organised to deliver treatment to particular groups.

In addition to improving implementation, future research should address gaps in knowledge. From our review, we highlight two key areas. The first is the hotly debated question about how to help trauma‐exposed children in the initial days or weeks posttrauma. Single‐session universal intervention appears unhelpful. Brief interventions for symptomatic children show some promise, but evidence is very scarce. There is an urgent need to develop early interventions that are acceptable to children and families, and helpful in reducing acute symptoms and preventing persistent PTSD. The challenge for the field is to determine the optimal ‘dose’ and content of intervention, and the optimal time point at which to deliver that intervention. These parameters are very likely to differ depending on the age of the trauma‐exposed child, and so flexibility in delivery will be needed. There is no evidence that early intervention is harmful for children, but given findings from some adult studies, it is important to test whether early interventions for children may cause harm. Investigating whether an early intervention has potential to impede natural recovery in the early stages after exposure would require inclusion of a no‐intervention comparison condition in a randomised controlled trial.

The second under‐researched area is in evaluating therapy for very young children. Developments of treatments for preschoolers have lagged behind the advances made with older children and adolescents. Inclusion in the DSM‐5 of developmentally sensitive diagnostic criteria for children 6 years and younger is encouraging, and may help to stimulate further work that can benefit this vulnerable group.

**Key practitioner messages**

- Trauma exposure is common among young people. By the end of adolescence, more than half of young people will have been exposed to at least one potentially traumatic event.
- Most trauma‐exposed young people do not develop PTSD: about 1 in 6 trauma‐exposed young people are likely to develop persistent PTSD.
- Assessment of trauma‐exposed young people should routinely employ valid and reliable interview and questionnaire measures of PTSD and other common problems.
- Trauma‐focused treatments (Trauma‐Focused CBT, Prolonged Exposure, Cognitive Therapy for PTSD) are effective in treating PTSD. Eye Movement Desensitization & Reprocessing (EMDR) is a promising treatment for PTSD in young people.
- There is no evidence that universally delivered early intervention is harmful for trauma‐exposed young people, but there is no robust evidence that such interventions reduce symptoms or prevent the onset of PTSD. Early interventions that are targeted to symptomatic young people appear more promising.

**Areas for future research**

- Psychometric studies will be needed to develop robust measures of PTSD symptoms and diagnosis according to the DSM and ICD frameworks.
- Further large‐scale evaluations of treatment effectiveness in nonspecialist community settings is needed.
- Further work on adapting and evaluating treatments for very young children with PTSD is required.
- Given limited service capacity in most health systems, new ways of delivering effective treatments are needed.
- Development and evaluation of early intervention for symptomatic trauma‐exposed children is needed.
